# Supplementary material for: Dual effects of NV-CoV-2 biomimetic polymer: An antiviral regimen against COVID-19
Source: PLoS One. 2022 Dec 30;17(12):e0278963. doi: 10.1371/journal.pone.0278963 (PMC9803160; doi:10.1371/journal.pone.0278963)
Supplement: Attachment — Submitted filename: PONE-Comments-9-06-22.doc [file pone.0278963.s001.doc]

PONE-D-22-20377
Dual Effects of NV-CoV-2 Biomimetic Polymer: An Antiviral regimen against COVID-19
PLOS ONE

Plos Comments:

5. Review Comments to the Author

Please use the space provided to explain your answers to the questions above. You may also include additional comments for the author, including concerns about dual publication, research ethics, or publication ethics. (Please upload your review as an attachment if it exceeds 20,000 characters)

**Reviewer #1:**

The manuscript “Dual Effects of NV-CoV-2 Biomimetic Polymer: An Antiviral regimen against COVID-19” was reviewed. The present study evaluates the efficacy and safety of a novel NV-387 polymer encapsulated remdesivir against coronavirus. The manuscript requires major revisions.

1) In the Introduction section, mortality or morbidity rates of coronavirus associated infections can be included.

**Au: Now included: shown in Line 54-59 and Blue highlighted.**

On 22 May 2020, there were 4,995,996 confirmed cases of SARS-CoV-2 including 327,821 deaths in 216 countries. The USA has reported the highest number of cases (1,525,186) and deaths (91,527) (mortality rate >12%) **[3],** and the number is increasing worldwide **[4]**. According to CDC reports, 94% of COVID deaths came from people with 2 to 3 comorbidities. 75% came from people with atleast 4 comorbidities **[5]***.*

2) The manuscript should be properly organized.

**Au: Now Done**

3) The titles and sequence of headings in the Methods and Results section should be same.

**Au: Now Done**

4) Moreover, some headings are in capital or upper case while others are in lower case. The heading pattern, font etc. should be same.

**Au: Now Corrected**

5) In the Methods section, the text appears to be primitive at some points. Furthermore, some text has been repeated; should be revised to avoid repetitions.

**Au: Now Corrected**

6) The authors are advised to elaborate the method of preparation and characterization of Dual function polymer.

**Au:** Now in the Method section a lot of information of our active material including it’s structure, formula and characterization are written (shown with Blue highlights).

In fact, this paper actually deals with the dual antiviral activities of NV-CoV-2 biopolymer with special reference to COVID-19. Lots of information of this biopolymer Model/Structure/Synthesis/Efficacy/Toxicity etc. are already published in many journals, Springer-Nature books and as a Press-release. All those references are cited here (Refs. 6-15, 26, 27, 29, 31, 50, 51]

7) In the Results section, some results, for example, subsection 2. In vitro antiviral activity of NV-CoV-2 should be revised to provide a clear understanding.

**Au: Now have written accordingly and shown with Blue highlights (Result section #2)**

8) The total number of Tables and Figures should be reduced; should not exceed 12. please consider merging of figures where appropriate.

**Au: Now done accordingly, 6 Figs and 6 Tables.**

9) In Figure 8, length bars should be included.

**Au: Now included**

10) In some figures / graphs, errors bars are missing; should be included.

**Au: Now included**

11) The manuscript should be revised to removed linguistic and typographical errors.

**Au: Now checked**
