# Supplementary material for: Dual effects of NV-CoV-2 biomimetic polymer: An antiviral regimen against COVID-19
Source: PLoS One. 2022 Dec 30;17(12):e0278963. doi: 10.1371/journal.pone.0278963 (PMC9803160; doi:10.1371/journal.pone.0278963)
Supplement: Attachment — Submitted filename: Rebuttal Letter-PONE-D-22-24874-101922.doc [file pone.0278963.s002.doc]

**Rebuttal Letter: Oct 19, 2022**

PLOS ONE Decision: Revision required [PONE-D-22-24874] -

**Journal requirements:**

When submitting your revision, we need you to address these additional requirements.

1.  Please ensure that your manuscript meets PLOS ONE's style requirements, including those for file naming. The PLOS ONE style templates can be found at

<https://journals.plos.org/plosone/s/file?id=wjVg/PLOSOne_formatting_sample_main_body.pdf>  and

<https://journals.plos.org/plosone/s/file?id=ba62/PLOSOne_formatting_sample_title_authors_affiliations.pdf>

**Au: Done accordingly**

2. We note that you have stated that you will provide repository information for your data at acceptance. Should your manuscript be accepted for publication, we will hold it until you provide the relevant accession numbers or DOIs necessary to access your data. If you wish to make changes to your Data Availability statement, please describe these changes in your cover letter and we will update your Data Availability statement to reflect the information you provide.

**Au: Data Availability statement: We want to reverse the statement what we had written earlier. No repository information is there that we have to submit later on. All the information are present in the manuscript.**

3. PLOS requires an ORCID iD for the corresponding author in Editorial Manager on papers submitted after December 6th, 2016. Please ensure that you have an ORCID iD and that it is validated in Editorial Manager. To do this, go to ‘Update my Information’ (in the upper left-hand corner of the main menu), and click on the Fetch/Validate link next to the ORCID field. This will take you to the ORCID site and allow you to create a new iD or authenticate a pre-existing iD in Editorial Manager. Please see the following video for instructions on linking an ORCID iD to your Editorial Manager account: <https://www.youtube.com/watch?v=_xcclfuvtxQ>

**Au: Orcid ID of Ashok Chakraborty (The first and Communicating Author)**

**0000-0002-0232-3036**

4. We note that you have included the phrase “data not shown” in your manuscript. Unfortunately, this does not meet our data sharing requirements. PLOS does not permit references to inaccessible data. We require that authors provide all relevant data within the paper, Supporting Information files, or in an acceptable, public repository. Please add a citation to support this phrase or upload the data that corresponds with these findings to a stable repository (such as Figshare or Dryad) and provide and URLs, DOIs, or accession numbers that may be used to access these data. Or, if the data are not a core part of the research being presented in your study, we ask that you remove the phrase that refers to these data.

**Au: “Data not shown”: Since those are not the core part of the Article, were removed.**

5. Your ethics statement should only appear in the Methods section of your manuscript. If your ethics statement is written in any section besides the Methods, please delete it from any other section.

**Au: “Ethics statement” was deleted from the other section except the “Methods” section.**
[Note: HTML markup is below. Please do not edit.]

**Reviewers' comments:**

**Reviewer's Responses to Questions**

**Comments to the Author**

1. Is the manuscript technically sound, and do the data support the conclusions?

The manuscript must describe a technically sound piece of scientific research with data that supports the conclusions. Experiments must have been conducted rigorously, with appropriate controls, replication, and sample sizes. The conclusions must be drawn appropriately based on the data presented.

Reviewer #1: Yes

Reviewer #2: Yes

2. Has the statistical analysis been performed appropriately and rigorously?

Reviewer #1: Yes

Reviewer #2: Yes

3. Have the authors made all data underlying the findings in their manuscript fully available?

The [PLOS Data policy](http://www.plosone.org/static/policies.action#sharing) requires authors to make all data underlying the findings described in their manuscript fully available without restriction, with rare exception (please refer to the Data Availability Statement in the manuscript PDF file). The data should be provided as part of the manuscript or its supporting information, or deposited to a public repository. For example, in addition to summary statistics, the data points behind means, medians and variance measures should be available. If there are restrictions on publicly sharing data—e.g. participant privacy or use of data from a third party—those must be specified.

Reviewer #1: Yes

Reviewer #2: Yes

4. Is the manuscript presented in an intelligible fashion and written in standard English?

PLOS ONE does not copyedit accepted manuscripts, so the language in submitted articles must be clear, correct, and unambiguous. Any typographical or grammatical errors should be corrected at revision, so please note any specific errors here.

Reviewer #1: Yes

Reviewer #2: Yes

5. Review Comments to the Author

Please use the space provided to explain your answers to the questions above. You may also include additional comments for the author, including concerns about dual publication, research ethics, or publication ethics. (Please upload your review as an attachment if it exceeds 20,000 characters)

**Reviewer #1:**

**Q#1: Results needed to be explained better. They have made statement on the basis of unpublished a number of times. Why they have not shown data.**

**Au: Those ‘data not shown’ part are the preliminary observation to standardize the materials and methods, and has little or no connection with the main paper, hence deleted from this revised version, also, the number of Figs remains within the limit.**

**Q#2: Discussion did not discuss results in very effective manner. Discussion does not detailed similar studies. They have not compared their results with other studies.**

**Au: Discussion part is now re-written accordingly, and English corrections were also done.**

**Reviewer #2:** The manuscript is well-written and well-presented. Besides, the study is well-designed. Only comment is that the authors claimed that “Remdesivir (RDV) is the only antiviral drug approved for COVID-19 therapy by the FDA”, **indeed molnupiravir is also approved by FDA as anti-COVID-19 drug**.

**Au:** Thanks for updating with the new Information, and accordingly **we have edited our Text (Please see, Lines 2-5); and also in the Discussion Section, Lines showed with Blue highlights**

**However, to exchange the information:**

LAGEVRIO™ (molnupiravir) **has not been approved,** **but has been authorized on December 23, 2021**, for emergency use by FDA, for the treatment of mild-to-moderate COVID-19 in adults who are at high risk for progression to severe COVID-19, including hospitalization or death, and for whom alternative COVID-19 treatment options authorized by FDA are not accessible or clinically appropriate *[https://www.lagevrio.com/patients/?utm]*

6. PLOS authors have the option to publish the peer review history of their article ([what does this mean?](https://journals.plos.org/plosone/s/editorial-and-peer-review-process#loc-peer-review-history)). If published, this will include your full peer review and any attached files.

If you choose “no”, your identity will remain anonymous but your review may still be made public.

**Do you want your identity to be public for this peer review?** For information about this choice, including consent withdrawal, please see our [Privacy Policy](https://www.plos.org/privacy-policy).

Reviewer #1: **Yes:** Muhammad Nouman Mughal

Reviewer #2: No
